# Acknowledgment to Reviewers of *Non-Coding RNA* in 2020

**DOI:** 10.3390/ncrna7010007

**Published:** 2021-01-25

**Authors:** Non-Coding RNA Editorial Office

**Affiliations:** MDPI AG, St. Alban-Anlage 66, 4052 Basel, Switzerland; ncRNA@mdpi.com

Peer review is the driving force of journal development, and reviewers are gatekeepers who ensure that *Non-Coding RNA* maintains its standards for the high quality of its published papers. Thanks to the cooperation of our reviewers, in 2020, the median time to first decision was 17 days and the median time to publication was 39.5 days. The editors would like to express their sincere gratitude to the following reviewers for their precious time and dedication, regardless of whether the papers were finally published:
Adriaens, CarmenJohnson, RoryAgnelli, LucaKang, Yang JaeAli, AliKawakami, JunjiAllegrucci, CinziaKlec, ChristianeAllmer, JensKlinge, Carolyn M.Anastasiadou, EleniKöhn, MarcelAndreassen, Rune J.Krasikova, AllaAndrés-León, EduardoKrause, Henry M.Bajan, SarahKristensen, Lasse S.Balatti, VeronicaLe Grice, Stuart F. J.Biagioli, MartaLee, Yong SunBiamonti, GiuseppeLeisegang, Matthias S.Bilia, Anna RitaLeucci, EleonoraBlandino, GiovanniLi, HaoboBracken, CameronLi, HowardBranlant, ChristianeLiang, TingmingBronisz, AgnieszkaLingner, JoachimBrown, Jessica A.LoVerde, PhilCalabrese, J. MauroMakalowska, IzabelaCarmichael, Gordon G.Marciniak, KatarzynaCerase, AndreaMartinez, ErnestChakrabarti, KausikMayer, Bryan T.Chang, SuhwanMeister, GunterCharchar, FadiMetere, AlessioChen, Ling-LingMilitello, GiuseppeCheng, HaiziMontes Resano, MartaChiu, Hua-ShengMorgan, MarcosChiu, NormanMorris, KevinChowdhury, Imran HussainMukhopadhyay, Arijit K.Clark, BrianMuxel, Sandra MarciaClezardin, PhilippeNakagawa, ShinichiConigliaro, AliceNigita, GiovanniCook, Atlanta G.Nociti, VivianaCoulouarn, CédricOgami, KoichiDajas-Bailador, FedericoOzer, Hatice GulcinDe Lencastre, AlexParchem, Ronald J.Deryusheva, SvetlanaPasini, LuigiDi Leva, GianpieroPayne, AnnetteDing, YiliangPrasanth, Kannanganattu V.Djuranović, SergejRasin, Mladen RokoDo, Duy NgocRaudsepp, TerjeDoody, GinaRayner, SimonDundr, MiroslavRenwick, NeilEnguita, Francisco J.Rodilla, VerónicaEslam, MohammedRomani, AndreaFabbri, MullerRoy, BhaskarFalzone, LucaSablina, AnnaFatkhudinov, Timur Kh.Schaefer, MatthiasFloris, MatteoSehgal, LalitGarcía-Ordás, María TeresaShelkovnikova, Tatyana A.Gaviraghi, MarcoShiina, MarisaGerber, AndréShyu, Ann-BinGonzález-Vallinas, MargaritaSmyth, RedmondGöringer, H. UlrichStafforst, ThorstenGorospe, MyriamStathopoulos, ConstantinosGradia, DanielaSztuba-Solinska, JoannaGuang, ShouhongTaguchi, YoshihiroGururajan, AnandTonge, DanielHappel, Christine M.Torres, Adrian GabrielHaspel, NuritUchida, ShizukaHendig, DorisValadkhan, SabaHenriksson, JohanWaghmare, SakharamHewitson, James P.Wellinger, Raymund J.Higgs, PaulWery, MaximeHueso, MiguelWilliams, JoannaHutchinson, John N.Yan, WeiIdogawa, MasashiYukawa, YasushiJohnson, AaronŻok, Tomasz

